# *MGMT* promoter methylation and 1p/19q co-deletion of surgically resected pulmonary carcinoid and large-cell neuroendocrine carcinoma

**DOI:** 10.1186/s12957-018-1413-7

**Published:** 2018-06-18

**Authors:** Lei Lei, Zhiming Jiang, Gu Zhang, Qiaoyuan Cheng, Hongyang Lu

**Affiliations:** 1 Zhejiang Key Laboratory of Diagnosis & Treatment Technology on Thoracic Oncology (lung and esophagus), Zhejiang Cancer Hospital, No. 1 East Banshan Road, Gongshu District, Hangzhou, 310022 People’s Republic of China; 2 Department of Pathology, Zhejiang Cancer Hospital, Hangzhou, 310022 People’s Republic of China; 3 Department of Health Food and Cosmetics, Zhejiang Institute for Food and Drug Control, Hangzhou, People’s Republic of China; 4 Department of Thoracic Medical Oncology, Zhejiang Cancer Hospital, Hangzhou, 310022 People’s Republic of China

**Keywords:** Pulmonary carcinoid, Large-cell neuroendocrine carcinoma, MGMT methylation, 1p/19q co-deletion

## Abstract

**Background:**

The response to temozolomide (TMZ) treatment in small-cell lung cancer (SCLC) correlated with O(6)-methylguanine -DNA methyltransferase (MGMT) promoter methylation. 1p/19q co-deletion within oligodendroglioma is a responsive predictor for TMZ. Currently, the status of *MGMT* promoter methylation and 1p/19q co-deletion in pulmonary carcinoid (PC) and large-cell neuroendocrine carcinoma (LCNEC) is not reported.

**Methods:**

Nine PC [two atypical carcinoids (AC), seven typical carcinoids (TC)] and six LCNEC patients were collected retrospectively. The pyrosequencing and fluorescence in situ hybridization were used to detect the MGMT promoter methylation and 1p/19q co-deletion in surgically resected specimens. Kaplan–Meier analysis was used to assess the rate of disease-free survival (DFS).

**Results:**

MGMT promoter methylation was found in two (2/6, 15.3%) LCNEC patients but not in any PC patients. Three (3/6, 50%) 1p and two (2/6, 33.3%) 19q single deletions were found in LCNEC patients. One 1p single deletion was found in AC patients. One (1/7, 14.3%) 1p and two (2/7, 28.6%) 19q single deletions were found in TC patients. After a median follow-up of 38 months, three LCNEC patients developed distant metastasis and one patient died of LCNEC disease. The DFS of PC patients was much longer than LCNEC patients (*χ*^*2*^ = 7.565, *P* = 0.006).

**Conclusions:**

MGMT promoter methylation and 1p/19q co-deletion might not be the ideal biomarkers for TMZ treatment in TC/AC patients. Thus, the detection of MGMT promoter methylation and whether it can be used as a medication for TMZ in LCNEC patients necessitates investigation. Furthermore, 1p deletion could be a negative prognostic factor for LCNEC patients.

**Electronic supplementary material:**

The online version of this article (10.1186/s12957-018-1413-7) contains supplementary material, which is available to authorized users.

## Background

Patients with pulmonary carcinoid (PC) tumors and low grade (typical carcinoid (TC)) and intermediate grade (atypical carcinoid (AC)) neuroendocrine tumors accounted for 1% of the lung cancer patients undergoing surgical treatment [1, 2]. PC shows common driver mutations from non-small-cell lung cancer (NSCLC) [3]. Surgery is the primary mode of treatment for stage I, II, or IIIA patients with TC or AC [4]. For the progressive pulmonary carcinoid tumors, systemic therapy with a combination of etoposide and platinum was still commonly used albeit with limited effects [5]. However, temozolomide (TMZ) as monotherapy or combined with capecitabine has been shown to be effective and well-tolerated [5, 6]. The combination of TMZ/cisplatin/docetaxel showed a remarkable response in one heavy treated metastatic pulmonary large-cell neuroendocrine carcinoma (LCNEC) patient [7].

The response to TMZ treatment in small-cell lung cancer (SCLC) may be associated with O(6)-methylguanine-DNA methyltransferase (MGMT) promoter methylation [8]. 1p/19q co-deletion is a positive response biomarker for TMZ treatment in oligodendrogliomas [9] and SCLC [10]. Unique profiles of aberrant methylation were observed in both SCLC and PC patients [11], and the potential PC patients who might benefit from TMZ are still to be ascertained [12]. However, whether *MGMT* promoter methylation or 1p/19q co-deletion existed in PC and LCNEC patients is yet unknown.

In order to elucidate the status of MGMT methylation and 1p/19q co-deletion in PC and LCNEC patients, two cases of AC, seven cases of TC, and six cases of LCNEC patients who underwent surgery were assimilated retrospectively and studied from the Zhejiang Cancer Hospital in China between 2008 and 2016.

## Methods

### Patients’ characteristics

Resected tumor samples were retrospectively collected from six LCNEC, seven TC, and two AC patients from the Zhejiang Cancer Hospital in China between 2008 and 2016. The pathological diagnosis was defined by the World Health Organization [13]. Patients were staged according to the eighth TNM classification for lung cancer [14]. Patient characteristics such as gender, age, the pathological type, stages, smoking history, and whether or not chemotherapy and/or radiotherapy was administered are described in Table 1. This study was approved by the Medical Ethics Committee of Zhejiang Cancer Hospital.

**Table 1:** Clinical features of surgically resected pulmonary carcinoid and LCNEC

Patient	Age	Sex	pTNM stage	Tumor type	Smoking status	Ki-67 (%)	Therapy
1	70	M	T2aN0M0,IB	LCNEC	No	40	S + C
2	57	M	T1cN0M1b, IV	LCNEC	Yes	30	S + C + R
3	56	F	T2aN0M0,IB	LCNEC	No	60	S + C
4	58	M	T3N1M0,IIIA	LCNEC	Yes	70	S + C + R
5	62	M	T1aN2M0,IIIA	LCNEC	Yes	60	S + C
6	48	F	T3N2M0,IIB	LCNEC	No	–	S + C + R
7	44	F	T2aN0M0, IB	TC	No	–	S
8	50	M	T1cN2M0,IIIA	AC	No	–	S + C + R
9	50	F	T2aN0M0, IB	AC	No	–	S
10	41	M	T1bN0M0,IA2	TC	No	–	S
11	61	M	T2bN0M0,IIA	TC	Yes	1	S
12	49	M	T1bN0M0,IA2	TC	No	8	S
13	63	F	T2bN1M0,IIB	TC	No	5	S
14	51	F	T1aN0M0,IA1	TC	No	1	S
15	53	M	T1bN0M0,IA2	TC	No	2	S

*LCNEC* large-cell neuroendocrine carcinoma, *TC* typical carcinoid, *AC* atypical carcinoid, *S* surgery, *C* postoperative chemotherapy, *R* postoperative radiotherapy

### Detection of *MGMT* promoter methylation and 1p/19q co-deletion

The methylation analysis of *MGMT* promoter was performed on bisulfite-converted DNA that was performed using a commercially available kit [EpiTect Bisulfite Kit (48)]. Subsequently, the bisulfite-converted DNA was utilized in the PCR reaction mix. The PCR conditions were as follows: firstly, 37 °C for 3 min and 95 °C for 3 min; secondly, 14 cycles of 95 °C for 15 s and 65 °C for 45 s; and finally, 26 cycles of 95 °C for 15 s and 60 °C for 45 s. Three microliters of amplification product was evaluated on AGE (agarose gel electrophoresis); 10 μL PCR product was combined with DNA fixing mix and shocked for 10 min at 1400 rpm (room temperature). Complete primer dilution with annealing buffer during this time. Bind PCR products with primers using PyroMark Q24 Vacuum Workstation (001790). Then sequence by PyroMark Q24 MDX (QIAGEN, Biotage, USA). Finally, results will be statistically analyzed using PyroMark Q24 Software (CpG mode). The 1p/19q co-deletion was detected in as reported previously by Lu et al. [10].

### Follow-up

The follow-up deadline was May 31, 2017. The median follow-up was 38 (11–112) months. Fourteen patients were alive, one LCNEC patient died, and no patients were lost to follow-up.

### Statistical analysis

The disease-free survival (DFS) was defined as the time from diagnosis to the progression of the disease or death, whichever is earlier or the last visit date. Kaplan–Meier analysis was used to assess the DFS). The log-rank test was used to estimate and compare the rate of survival. All statistical analyses were carried out on an intention-to-treat basis using the SPSS 15.0 software package (SPSS Inc., Chicago, IL, USA).

## Results

The median age was 50 (41–63) years for TC/AC and 58 (48–70) years for LCNEC patients. The majority of the patients presented a postoperative mass size of T1–2, regional lymph node metastasis of N0–1, and no adjuvant treatment after surgery except in one patient (T1cN2M0, stage IIIA) who had received 4 cycles of etoposide and platinum combination chemotherapy and local radiotherapy postoperatively. One pulmonary LCNEC patient with de novo IV stage underwent excision of brain metastatic lesion and chemotherapy with 2 cycles of etoposide with cisplatin and 2 cycles of docetaxel with cisplatin and the palliative pulmonary radiotherapy. All the other PC patients received no adjuvant chemotherapy or radiotherapy, while the pulmonary LCNEC patients received combined chemotherapy containing platinum regimen as adjuvant chemotherapy, and three patients also accepted the adjuvant radiotherapy. The de novo stage IV LCNEC patient progressed in the brain and lung only 1 year after the palliative cerebral metastatic tumor excision and systemic therapy. The other two LCNEC patients developed distant metastases (one case in cervical vertebrae, one case in brain), and only one patient with cervical vertebrae metastasis died. None of the TC/AC patients showed a relapse or metastasis after surgery during the median follow-up of 51 (11–112) months (Table 2). The DFS of PC patients was much better than that of LCNEC patients (*χ*^*2*^ = 7.565, *P* = 0.006) (excluding the patient of de novo stage IV LCNEC patient).

**Table 2:** MGMT promoter methylation, 1p/19q deletion, and clinical outcome of surgically resected pulmonary carcinoid and LCNEC

Patient	1p Del	19q Del	MGMTMet	Metastasis (ML)	DFS (mon)	OS (mon)
1	No	Yes	No	Yes (TV)	10	36
2	Yes	No	No	Yes (B)	29	29
3	No	Yes	Yes	No	22	22
4	No	No	No	No	41	41
5	Yes	No	Yes	Yes (B)	12	44
6	Yes	No	No	Yes (CV; DOD)	9	34
7	No	No	–	No	112	112
8	No	No	No	No	107	107
9	Yes	No	No	No	101	101
10	No	Yes	–	No	84	84
11	No	No	No	No	37	37
12	Yes	No	No	No	31	31
13	No	No	No	No	51	51
14	No	No	–	No	38	38
15	No	Yes	No	No	11	11

*LCNEC* large-cell neuroendocrine carcinoma, *Del* deletion, *Met* methylation, *ML* metastatic lesions when the first metastasis occurred, *B* brain, *TV* thoracic vertebra, *CV* cervical vertebra, *DOD* dead of disease, *DFS* disease-free survival, *OS* overall survival

**Fig. 1:**
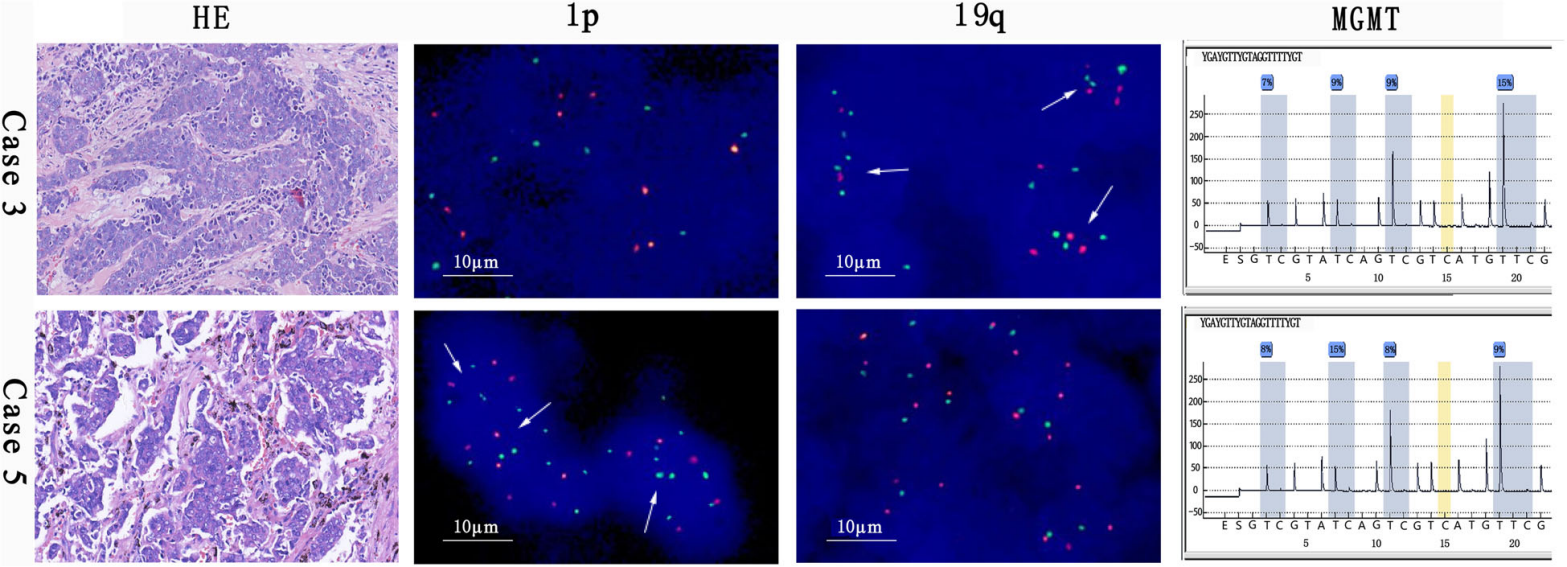
The results of MGMT promoter methylation and 1p/19q deletion for cases 3 and 5. Positive result for MGMT promoter methylation by pyrosequencing. The quantification results of ploidy (O:G): 1p was 14% and 19q was 22% in case 3; 1p was 50% and 19q was 4% in case 5. The arrows indicate a ratio of O:G < 0.87 in the cells. Magnification for HE is × 200. G green, O orange, he hematoxylin–eosin

The MGMT promoter methylation was found in two (2/6, 15.3%) LCNEC patients (Fig. 1). Three (50%) 1p single deletion and two (33.3%) 19q single deletions were found in LCNEC patients (Fig. 1 and Additional file 1: Figure S1). 1p single deletion was found in one AC patient. Another one (1/7, 14.3%) 1p and two (2/7, 28.6%) 19q single deletions were found in TC patients. Three PC patients failed MGMT promoter methylation testing because of the quality of specimens. No MGMT promoter methylation was found in the remaining TC/AC patients. 1p/19q co-deletion was not found in all patients (Table 2).

## Discussion

Dixon et al. reported that 75.5% of PCs were females, and the median age was 60.7 years, and 53.1% were smokers [15]. Ayadi-Kaddour et al. reported 56 men and 59 women with a mean age of 43.73 years in a cohort of 100 TC and 15 AC patients [4]. We found that 44.4% of PCs were females, TCs were more than ACs (77.8 vs. 22.2%), and only one male patient was a smoker (11.1%) in nine PCs with a median age of 50 (41–63) years. The clinical characteristics of patients in the current study were similar to those reported previously [4, 15]. The outcome of PC patients was favorable after surgery, and the factors affecting the survival included tumor size, nodal status, distant metastasis, and typical/atypical tumor [4, 16]. TCs have a better 5-year survival rate and lower incidence of metastasis than ACs after surgery [17]. Somatostatin analogs are considered as the first-line treatment of carcinoid syndrome, especially in some low-grade TCs and ACs. Systemic chemotherapy is recommended for progressive and unresectable PCs. Compared to other palliative chemotherapies, TMZ has similar anti-tumor efficacy but is well-tolerated and could be used in metastatic brain PCs [5].

LCNEC was first introduced by Travis in 1991 and defined as a variant of large-cell carcinoma by the World Health Organization (WHO) from 1999 to 2004 [18–20]. When sufficient specimens are available for the diagnosis of LCNEC, surgical specimens should be the first choice than biopsy or cytology specimens [21]. LCNECs comprise of 1.6–3% resectable lung cancers [22, 23] that are common in males [24]. Reportedly, the 5-year overall survival varies between 13 and 57% for LCNECs [23–25]. In contrast to PCs, LCNECs are similar to SCLCs, always related with smoking history [26]. In the current study, the median age of six LCNECs was 58 (48–70) years, half of them were smokers, and two were female patients. The postoperative adjuvant chemotherapy regimen was a platinum-containing regimen consisting of two drugs of 4 cycles according to the recommendations of the NCCN guidelines. After the median follow-up of 35 (29–44) months, four out of six patients developed distant metastasis and one died due to the disease. Moreover, two had vertebral bone metastasis, while another two had brain metastasis. The median DFS was worse in LCNEC patients than in PC patients (12 vs. 51 months, *P* = 0.006). No standard chemotherapy for advanced LCNEC is yet recommended. Compared to SCLC, LCNEC responded with inferior efficacy to first-line cisplatin and irinotecan chemotherapy as assessed by the response rate (RR) and median survival time (MST) (RR 46.7 vs. 80%, respectively, *P* = 0.0823; MST 12.6 vs. 17.3 months, respectively, *P* = 0.047) [27]. The prognosis of LCNEC in neuroendocrine lung carcinoma seemed to occur between PC and SCLC and rather prone to the poor prognosis of SCLC.

Advanced lung cancer remains to be a catastrophic disease, partly due to the high incidence of brain metastasis [28]. TMZ is not much of a blood-brain barrier penetrating cytotoxic drug that is rather proven to be effective in malignant brain metastasis treatment [29, 30]. TMZ-based chemotherapy is recommended as first- or second-line treatment for TC or AC with negative somatostatin receptor (SSR) and rapid progression [31]. In our previous report, 33 SCLC specimens obtained from surgery were collected retrospectively and analyzed by high-resolution melting (HRM) analysis and methylation-specific polymerase chain reaction (MSP); MGMT promoter methylation was detected in 17 patients (51.5%) [32]. 1p/19q co-deletion was found in three patients, who survived after 58, 50, and 30 months of follow-up comprising of 32 SCLC resected specimens, thereby indicating a good prognostic factor in SCLC [10]. Several studies reported the use of TMZ in treating LCNEC patients [7]. Toyooka et al. demonstrated that bronchial carcinoids had lower frequencies of MGMT methylation than SCLC; however, the difference is not significant [11]. Three PC patients failed in MGMT promoter methylation testing owing to poor quality of specimens, and the remaining had negative results. Consequently, whether the frequency of MGMT promoter methylation is lower as compared to SCLC is yet to be elucidated. Currently, no study focusing on MGMT promoter methylation in LCNEC or 1p/19q deletion in both PC and LCNEC patients has been reported. Despite the small number of patients incapable of survival analysis, we deduced the following: firstly, MGMT promoter methylation was detected in two LCNEC but not TC/AC patients, which might be correlated to the better outcome of TC/AC than LCNEC patients. Secondly, three metastatic LCNEC patients carried the deletion of 1p, thereby designating it as an unfavorable prognostic factor for LCNEC patients.

## Conclusions

Although the number of cases in this study was small, the specimens used for marker detection and pathological diagnosis originated from surgical specimens with reliable results. To the best of our knowledge, this is the first report about the exploratory detection of MGMT and chromosome 1p/19q deletion on three rare pulmonary neuroendocrine carcinomas in addition to SCLC. MGMT promoter methylation and 1p/19q co-deletion may not be an ideal biomarker for TMZ treatment in TC/AC patients. Furthermore, the MGMT promoter methylation could be used as medication instruction for TMZ in LCNEC patients. The deletion of 1p might be a negative prognostic factor for LCNEC, and a prolonged follow-up and large sample size would be valuable for further verification.

## Additional file

Additional file 1: **Figure S1.** Positive control and negative control for 1p and 19q. 1p was 52% and 19q was 66% in positive control; 1p was 5% and 19q was 3% in negative control. (JPEG 188 kb)
