# Supplementary figures and images for: MGMT promoter methylation and 1p/19q co-deletion of surgically resected pulmonary carcinoid and large-cell neuroendocrine carcinoma
Source: World J Surg Oncol. 2018 Jun 18;16:110. doi: 10.1186/s12957-018-1413-7 (PMC6007073; doi:10.1186/s12957-018-1413-7)

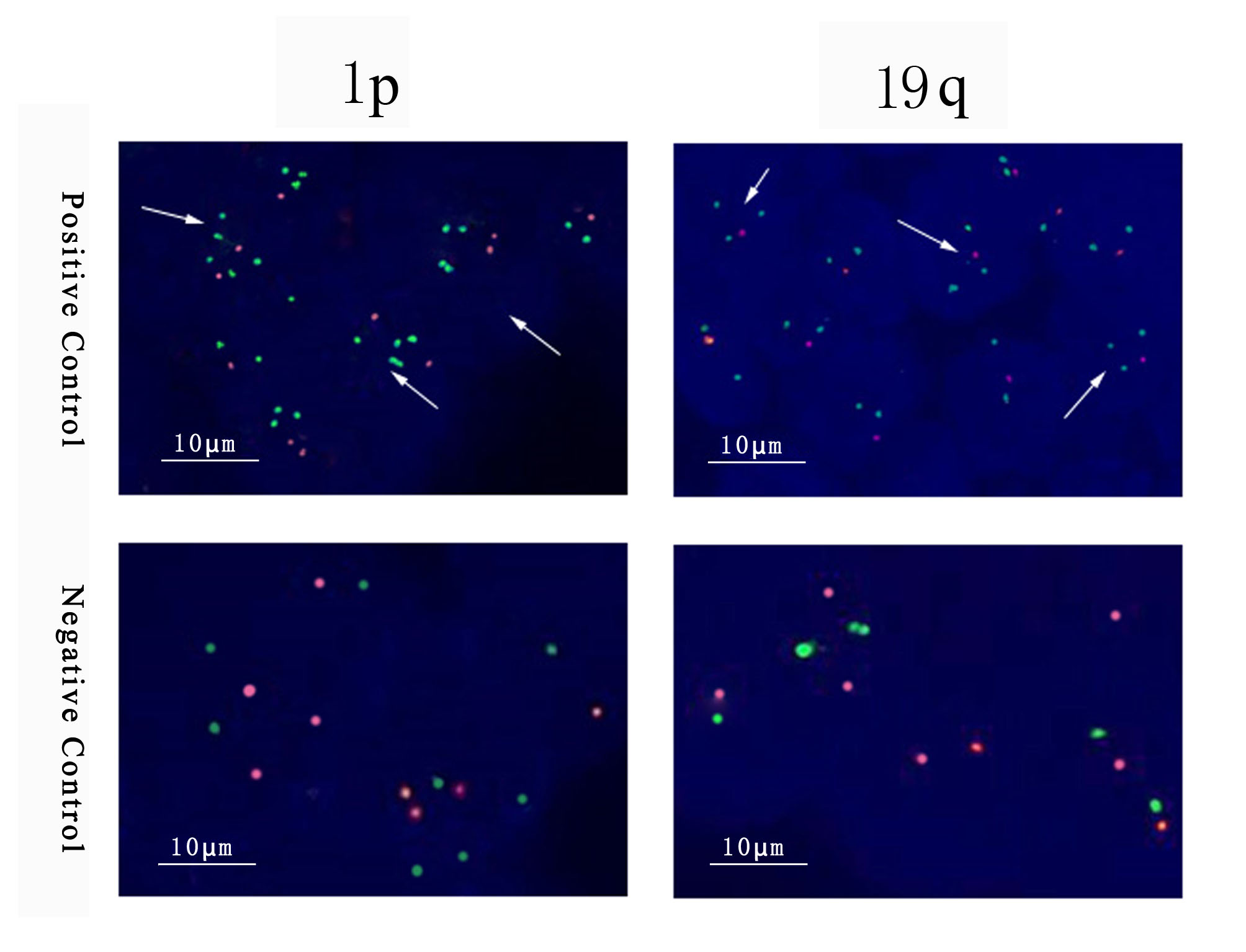

Supplement: Supplementary file 1 — Figure S1. Positive control and negative control for 1p and 19q. 1p was 52% and 19q was 66% in positive control; 1p was 5% and 19q was 3% in negative control. (JPEG 188 kb) [file 12957_2018_1413_MOESM1_ESM.jpeg]
